# Double-dose investigation of aflibercept in neovascular age-related macular degeneration (DIANA): a real-world study

**DOI:** 10.1186/s12886-024-03476-9

**Published:** 2024-05-17

**Authors:** Min Zhang, Xing Liu, Yuanyuan Gong, Tianwei Qian, Hao Zhou, Yimin Wang, Jiali Wu, Xiaodong Sun, Suqin Yu

**Affiliations:** 1 Department of Ophthalmology, Shanghai General Hospital, Shanghai Jiao Tong University School of Medicine, Wujin Road 85, Hongkou District, Shanghai, China; 2 Quanzhou Women’s and Children’s Hospital, Fujian, China; 3 National Clinical Research Center for Eye Diseases, Shanghai, China; 4 Shanghai Key Laboratory of Ocular Fundus Diseases, Shanghai, China; 5 Shanghai Engineering Center for Visual Science and Photomedicine, Shanghai, China; 6 Shanghai Engineering Center for Precise Diagnosis and Treatment of Eye Diseases, Shanghai, China

**Keywords:** Neovascular age-related macular degeneration, Anti-VEGF treatment, Aflibercept, High-dose, Real-world study

## Abstract

**Background:**

To investigate the clinical effects of double-dose (4 mg) aflibercept treatment in neovascular age-related macular degeneration (nAMD), compared with the standard-dose (2 mg) treatment.

**Methods:**

A total of 108 eyes from 97 patients with nAMD and received intravitreal aflibercept 2 mg and/or 4 mg treatment were retrospectively reviewed. The changes of central macular thickness (CMT)/ pigmental epithelium detachment height and the recurrence rate of exudation during the 12-month follow-up were compared between the 2 mg group and the 4 mg group. Self-control comparisons (2 mg switch to 4 mg) were also made between two regimens.

**Results:**

Compared with the 2 mg group, tendencies of lower intraretinal fluid incidence and more CMT reduction were observed in the 4 mg group. The later one was also observed when eyes switching from 2 mg to 4 mg regimen. The median remission interval was 5 months in the 4 mg group, 2 months longer than the 3 months in the 2 mg group (*P* = 0.452). Injections needed in the 4 mg group were 3.644 ± 1.670, less than the 4.286 ± 2.334 injections in the 2 mg group within 12 months as well (*P* = 0.151). However, no associated vision benefits were gained from the double-douse regimen. No markedly increased-intraocular pressure events, or other adverse events were found in two groups.

**Conclusions:**

Compared to the aflibercept 2 mg treatment in nAMD, tendencies of anatomic gains and relieving treatment burden were brought by the aflibercept 4 mg treatment. This study may have additional importance, given the further application of high-dose aflibercept in real-world settings.

**Supplementary Information:**

The online version contains supplementary material available at 10.1186/s12886-024-03476-9.

## Background

Neovascular age-related macular degeneration (nAMD) is a leading cause of irreversible visual loss in elderly people. Although the introduction of intravitreal vascular endothelial growth factor (VEGF) injection greatly improved the visual prognosis in patients with nAMD, a notable subpopulation with resistant macular neovascularization (MNV) can be easily found [1]. In addition, frequent injections and follow-up visits are quite inconvenient and burdensome for patients with nAMD, especially during the COVID-2019 pandemic [2]. Strategies have to be developed for a further increase in anti-VEGF efficacy to nAMD.

High-dose treatment is one of the most frequently employed strategies. Visual and anatomic gains were achieved with 2.0 mg ranibizumab in ranibizumab-0.5-mg-resistant nAMD in the SAVE study [3] and in the LAST study [4]. Although the HARBOR study reported no superiority of ranibizumab 2.0 mg to ranibizumab 0.5 mg in treatment-naïve patients with nAMD, the DoDo trial demonstrated trends toward higher efficacy with less frequent injections using ranibizumab 1.0 mg compared with 0.5 mg for treatment-naïve nAMD [5]. Therefore, the efficacy of high-dose ranibizumab treatment in resistant MNV and its advantages in treatment-naïve nAMD were well recognized.

After pegaptanib, bevacizumab, and ranibizumab, aflibercept, a fully recombinant fusion protein of domains from human VEGF receptor 1 and 2, was created to bond more strongly with VEGF [6]. In the pivotal VIEW 1 and VIEW 2 studies [7], all regimens of aflibercept, including 0.5 mg and 2 mg, showed non-inferiority to monthly ranibizumab 0.5 mg. Although aflibercept 2 mg treatment regimens currently in use recommend intravitreal injections at two-month intervals with monthly monitoring [8], about 45% eye needed to be escalated to 2 mg monthly injection [9]. Monthly intravitreal aflibercept 4 mg was reported to be an effective treatment for patients with resistant nAMD [10], indicating the rationale for higher doses of aflibercept treatment in it.

In our clinical practice, the aflibercept double-dose regimen has become an empirical treatment. Ever since aflibercept became commercially available, a number of nAMD patients, either treatment-naïve or treated with other anti-VEGF agents before, received aflibercept treatment under its standard-dose regimen. However, some of them switched to the double-dose regimen due to poor or incomplete response. Gradually, some patients even started with double-dose regimen. Although this empirical strategy has been employed, its additional anatomic gains and treatment burden relieves have not been confirmed yet. Therefore, this retrospective study was designed to evaluate the clinical effects of double-dose aflibercept on patients with nAMD in the real-world practice. Patients who received standard-dose (2 mg) and/or double-dose (4 mg) aflibercept were reviewed. Visual and anatomic gains, injection frequencies, resolution intervals, and adverse events were compared. A self-comparison before and after dosage change was conducted in some patients as well.

## Methods

### Study design

The primary objectives of the study were to compare the effect of intravitreal 4 mg versus 2 mg aflibercept injection on central macular thickness (CMT)/ pigmental epithelium detachment (PED) height and to assess the resolution of intraretinal fluid (IRF)/subretinal fluid (SRF) in patients with MNV associated with nAMD in a real-world setting. The secondary objectives were to assess the effect of double-dose aflibercept on BCVA, remission/recurrence in one year under the PRN regimen, when compared to the standard-dose treatment.

Double-dose Investigation of Aflibercept in Neovascular Age-related macular degeneration (DIANA) was a retrospective, single-centered, real-world study. All patients provided written informed consent to receive treatment for each intravitreal injection. The selection of aflibercept doses were made based on patients’ own decisions, and specific consent for double-dose treatment were taken from corresponding patients. All data analyzed were anonymized and de-identified.

### Study population

Clinical records were retrospectively searched for all participants, with typical nAMD as well as PCV, receiving aflibercept therapy (both standard-dose and double-dose) from July 23, 2018, to January 12, 2022. Typical AMD refers to type 1, 2 and 3 MNV in nAMD, but only type 1 and 2 MNV cases were included in this study.

Patients were eligible for the DIANA trial if they were aged 50 years or older and met the following inclusion criteria for the study eye: (1) Active baseline MNV lesion(s), indicated by SRF and/or IRF on OCT images. (2) No intravitreal injections or systematic administration of anti-VEGF agents for at least 3 months before the study baseline of 2 mg or 4 mg. Table S1 provided an example of the treatment schedule from one patient, illustrating the definition of “at-least-3-month-interval before the study baseline”. (3) Baseline visual acuity LogMAR ≤ 2 (20/2000 Snellen equivalent). (4) Baseline intraocular pressure (IOP) ≤ 20 mmHg. (5) Followed up for at least 1 year. Specifically, a history of nAMD in their fellow eyes was allowed. Study eye with baseline subretinal fibrosis was not excluded. Subretinal fibrosis refers to well-defined hyperreflective material between the neurosensory retina and the Bruch membrane on OCT, corresponding to the yellowish pallor of the lesion on color fundus photography [11].

Key exclusion criteria (for the study eye) were: (1) A history of vitrectomy surgery, treatment with photodynamic therapy with verteporfin, additional external beam radiation therapy, or transpupillary thermotherapy. (2) Previous intravitreal non-anti-VEGF drug delivery. (3) Presence of non-nAMD caused macular edema, such as diabetes or retinal vein occlusion. (4) Presence of non-nAMD caused macular neovascularization, such as pachychoroid induced or myopic choroidal neovascularization. (5) A history of glaucoma. (6) Massive subretinal hemorrhage, defined as a high blood volume not limited to the vascular arcades or leading to a hemorrhagic retinal detachment [12].

As the dose were selected based on patients’ own decisions, the scenario of almost 2/3 recruited patients in the 4 mg group reflected patients’ simple wish: the higher dose, the stronger effect. To reduce the potential selection bias in this process, we recruited not only treatment-naïve patients, but also patients with anti-VEGF treatment history, even those experiencing both aflibercept 2 mg treatment and 4 mg treatment. Futhermore, to make sure one-to-one matching in self-control comparisons, only eyes with the same follow-up time point in different dose groups were included (if multiple time points were available, the longest time point was selected).

### Treatment, follow-ups, and assessments

The aflibercept administrated in patients was commercially available (Eylea, Bayer Consumer Care AG, Basel, Switzerland for use outside of the USA). Patients received aflibercept 2 mg treatment had 0.05 mL aqueous solution delivered into the vitreous cavity, and patients received 4 mg treatment had 0.10 mL. Both treatments had the concentration of aflibercept at 40 mg/mL.

All treatment-naïve cases received three loading injections first. Thereafter, the monthly injection was administrated until a dry macula was achieved. Additional injections were then resumed in case of disease activity under the PRN regimens. For previously-treated patients, the PRN regimen was also employed. Under this regimen, all patients were followed every month, if not interrupted by the COVID-19 quarantine. The active lesion was defined as the presence of macular exudation, including IRF, SRF, or new bleeding. Vision criteria for retreatment were not employed. If serous/neovascular PED persisted but was stable for consecutive visits, the injections could be skipped. Instead, even with no exudation, injections were resumed if PED height reduction was observed after the last injection. In addition, no patients in this study received photodynamic therapy, due to the commercial unavailability of verteporfin since 2019.

At each visit, patients underwent measurement of vital signs (mainly heart rate and blood pressure), IOP testing, and examination of the anterior and posterior segments. Major illness or surgery during the follow-ups were also documented. OCT (Spectralis; Heidelberg Engineering) and color fundus imaging (Visucam 200 or CLARUS 500; Carl Zeiss Meditec AG), were also performed at each follow-up.

To investigate the clinical effects of aflibercept 4 mg treatment, the demographic data (age, gender), medical history, and visual performances of enrolled patients were reviewed and collected. Specifically, the medical history included IRF/SRF/CMT/PED height at baseline and each visit, number of intravitreal aflibercept 2 mg/4 mg injections, and follow-up duration. The CMT was measured as the central foveal distance from the inner limiting member to the Bruch’s membrane on the structural OCT images using the instrument’s calipers. Similarly, the maximal PED height (shortened as PED height) was measured from the top of the detached RPE to the Bruch’s membrane on all the patients in the same way. CMT/PED height changes were calculated by CMT/PED height at each follow-up visit minus baseline CMT/PED height.

Recurrence was defined as either IRF, SRF, or new bleeding after a dry condition according to macular anatomical status. Remission intervals were recorded from the injection visit to the visit one month before the first following visit with recurrence (**Formula 1**). For eyes with IRF/SRF at the one-month visit after the injection, remission should not be marked (or the remission interval was zero). Study eyes experiencing several remission/recurrences had several remission intervals. Therefore, the longest remission interval and the cumulative remission interval during the 1-year follow-up were calculated for these eyes.

 *Remission interval (months)*

*=the correspoinding first exudative visit (recurrence)*

*-the injectin visit-1 month*

### Statistical analyses

A total of 108 eyes from 97 patients with nAMD, including polypoidal choroidal vasculopathy (PCV), were treated with aflibercept 2 mg or 4 mg. Anatomic and clinical features during the first 12-month follow-ups were firstly compared between two groups. In this part, patients who switched doses only had their 4 mg follow-up analyzed. Instead, the two-period treatment of these 15 patients (experiencing a switch from the 2 mg treatment to the 4 mg treatment) were both used in the subsequent self-control analysis.

For the demographic and baseline characteristics, the normality of continuous variables was tested, and those following the normal distribution were expressed as mean ± standard deviation (SD). Student *t*-test was used to compare the differences between the aflibercept 2 mg group versus the 4 mg group. Parameters that did not conform to normal distribution were expressed as median (interquartile range), and the Mann-Whitney U test was used to compare between the two groups. Categorical data were represented by n (%), and the Chi-square test was used for comparisons.

The following parameters were measured repeatedly due to different eyes in one patient and different time points during the follow-ups [monthly]: IRF, SRF, CMT, and PED height. The Generalized Estimating Equation (GEE) was used to detect the risk factors of exudative recurrence. The linear regression model was used as the connection function for the continuous outcome (CMT/PED height/remission intervals), and the binary Logistic regression model was used as the connection function for the dichotomous outcomes (IRF/SRF). The difference between the two dose groups (2 mg versus 4 mg) was the main outcome of the model, and baseline characteristics (including age, gender, subtype of the treated eye [typical AMD or PCV], baseline visual acuity of study eye [LogMAR], the previous number of anti-VEGF treatments [treatment-naïve marked as zero], baseline CMT/ baseline PED height, etc.) were used as adjustable covariates.

The time-event outcome (the first recurrence during the follow-ups) was analyzed by survival analysis. Recurrence time was defined as the time interval from the first injection to the first recurrence. The Kaplan-Meier (K-M) method was used for the survival curve, and the Cox regression model was used to compare the hazard Ratio (HR) and 95% Confidence interval (CI) between different dose groups. Baseline characteristics (same as mentioned above) were included as adjustable covariates.

SPSS software (IBM SPSS Statistics for Windows, version 25.0, IBM Corp., Armonk, N.Y., USA) was used for statistical analyses. Test level α was set at 0.05. *P* value less than 0.05 was considered to be statistically significant.

## Results

There were 35 eyes from 34 patients receiving aflibercept 2 mg treatment and 73 eyes from 63 patients receiving aflibercept 4 mg treatment. In this retrospective study, compared with eyes in the 2 mg group, those in the 4 mg group had thinner baseline PED height (364.000 ± 215.861 μm versus 264.260 ± 180.315 μm, *P* = 0.013) but more cases receiving anti-VEGF injections before (37.143% versus 64.384%, *P* = 0.007; Table 1).

**Table 1:** Demographics and general characteristics of enrolled eyes with neovascular age-related macular degeneration (nAMD)

Mean ± SD or *n* (%)	2 mg	4 mg	*P* value
Age (years old)	70.771 ± 9.974	71.274 ± 9.647	0.805
Gender (male, %)	23 (65.714%)	41 (65.753%)	0.997
Subtype			
Typical AMD	22 (62.857%)	54 (73.943%)	0.253
PCV	13 (37.143%)	17 (23.288%)	
Baseline visual acuity (LogMAR)^#^	0.757 ± 0.400	0.656 ± 0.398	0.260
LogMAR > 0.3 (n, %)	27 (77.143%)	56 (76.712%)	0.961
LogMAR > 0.5 (n, %)	23 (65.714%)	41 (56.164%)	0.349
Baseline CMT (µm)	419.543 ± 234.597	375.987 ± 238.210	0.374
Baseline PED height (µm)	364.000 ± 215.861	264.260 ± 180.315	0.013
Medical history (yes, %)	13 (37.143%)	47 (64.384%)	0.007
Previous anti-VEGF injections	8.045 ± 7.286	11.269 ± 9.119	0.188
Number of injections (within 12 months)	4.286 ± 2.334	3.644 ± 1.670	0.151

^#^There were two missing visual acuity values, both in the 4 mg group

AMD = age-related macular degeneration, PCV = polypoidal choroidal vasculopathy. PCV is a neovascular AMD subtype, and here typical AMD refers to type 1, 2 and 3 macular neovascularization in neovascular AMD

CMT = central macular thickness, PED = pigmental epithelium detachment

### Anatomic features on OCT

Controlled by age, gender, subtype, baseline CMT, baseline PED height, and medical history (treatment-naïve or previously receiving anti-VEGF treatment), the relationships between OCT features and aflibercept doses were analyzed by GEE models. Although no statistical significance was found, a tendency of lower IRF incidence (adjusted odds ratio [OR] = 0.433, 95% confidence interval [CI] = 0.183 to 1.022, *P* = 0.056), but higher SRF incidence (OR = 1.539, 95% CI = 0.755 to 3.139, *P* = 0.236, Table 2) were detected in the 4 mg group.

**Table 2:** Optical coherence tomography (OCT) features in two dose groups during the first 12 months

Outcomes	Dose	OR (95% CI)	*P* value
Either IRF or SRF^#^	2 mg	1.000 (Reference)	-
	4 mg	0.934 (0.493 to 1.770)	0.835
IRF only^#^	2 mg	1.000 (Reference)	-
	4 mg	0.433 (0.183 to 1.022)	0.056
SRF only^#^	2 mg	1.000 (Reference)	-
	4 mg	1.539 (0.755 to 3.139)	0.236

Outcomes	Dose	Regression coefficient (95% CI)	*P* value
CMT changes (µm)^*^	2 mg	0.000 (Reference)	-
	4 mg	-25.242 (-90.024 to 39.539)	0.445
PED height changes (µm)^**^	2 mg	0.000 (Reference)	-
	4 mg	61.726 (-2.308 to 125.761)	0.059
Longest remission interval (months)^#^	2 mg	0.000 (Reference)	-
	4 mg	-0.207 (-1.428 to 1.014)	0.740
Cumulative remission interval (months)^#^	2 mg	0.000 (Reference)	-
	4 mg	0.394 (-0.135 to 0.922)	0.144

OR = odds ratio, CI = confidence interval, CMT = central macular thickness, PED = pigmental epithelium detachment

#Generalized Estimating Equation (GEE) model, controlled by age, gender, subtype, baseline CMT, baseline PED height, and medical history (treatment-naïve or previously receiving anti-VEGF treatment)

^*^GEE model, controlled by ages, gender, subtype, baseline PED height, and medical history; CMT changes = follow-up CMT minus baseline CMT

^**^GEE model, controlled by ages, gender, subtype, baseline CMT, and medical history; PED height changes = follow-up PED height minus baseline PED height

CMT or PED height beyond measurement was marked as missing values

**Fig. 1:**
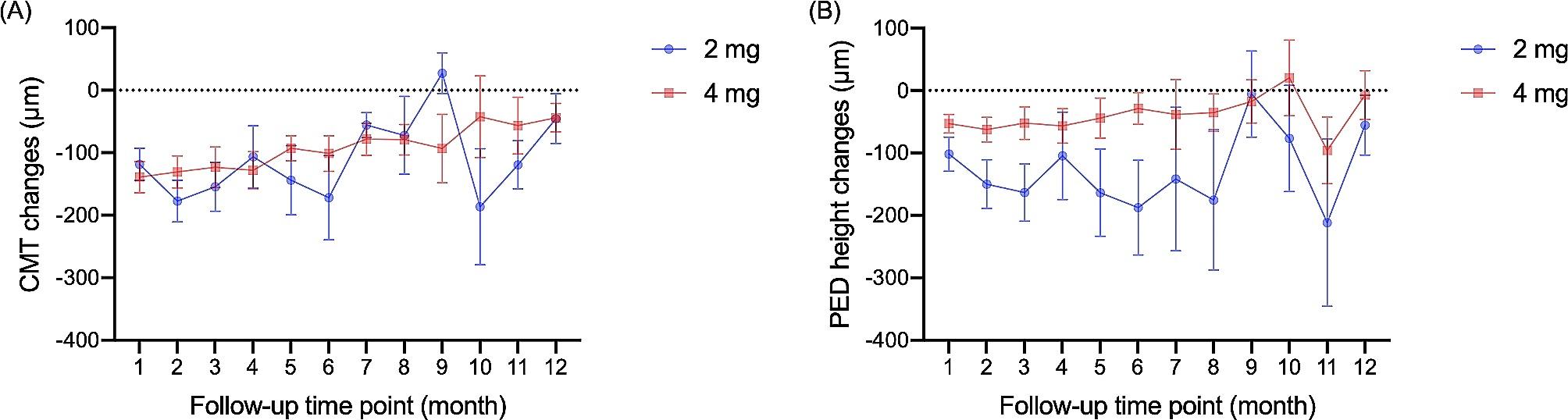
Central macular thickness (CMT) and pigmental epithelium detachment (PED) height changes during the first 12 months (**A**) CMT changes during the follow-ups (**B**) PED height changes during the follow-ups Data were presented as mean ± standard error

Both aflibercept 2 mg and 4 mg treatments effectively reduced the CMT and PED height (Fig. 1). Great variations of both CMT and PED height during the follow-ups were detected in the 2 mg group, and both changes were largely reduced at the 9th month in the 2 mg group. Meanwhile, the changes in the 4 mg group steadily went down (the value of changes going to zero, Fig. 1). Effective PED height reduction in the 4 mg group reached zero in the 10th month, but the CMT reduction in the 4 mg group was kept during the whole 12-month-follow-ups. Compared with the 2 mg group and with covariates controlled, more CMT reduction was observed in the 4 mg group (regression coefficient = -25.242 μm, 95% CI = -90.024 μm to 39.539 μm, *P* = 0.445, Table 2), while less PED height reduction was found in the 4 mg group (regression coefficient = 61.726 μm, 95% CI = -2.308 μm to 125.761 μm, *P* = 0.059).

### Visual acuity

There were 98 eyes from 89 patients included in the visual acuity analysis. Before the 8th month, the visual acuity of both groups had minor fluctuations around a state of equilibrium. After the 8th month, the visual acuity in the 2 mg group still got improved but that in the 4 mg group got worse (Figure S1). Controlled by ages, gender, subtype, baseline CMT, baseline PED height, and medical history, the aflibercept 4 mg treatment did not have an advantage over aflibercept 2 mg treatment in visual preservation (adjusted OR = -0.133, 95% CI = -0.227 to -0.039, *P* = 0.006).

### Remissions and recurrences

**Fig. 2:**
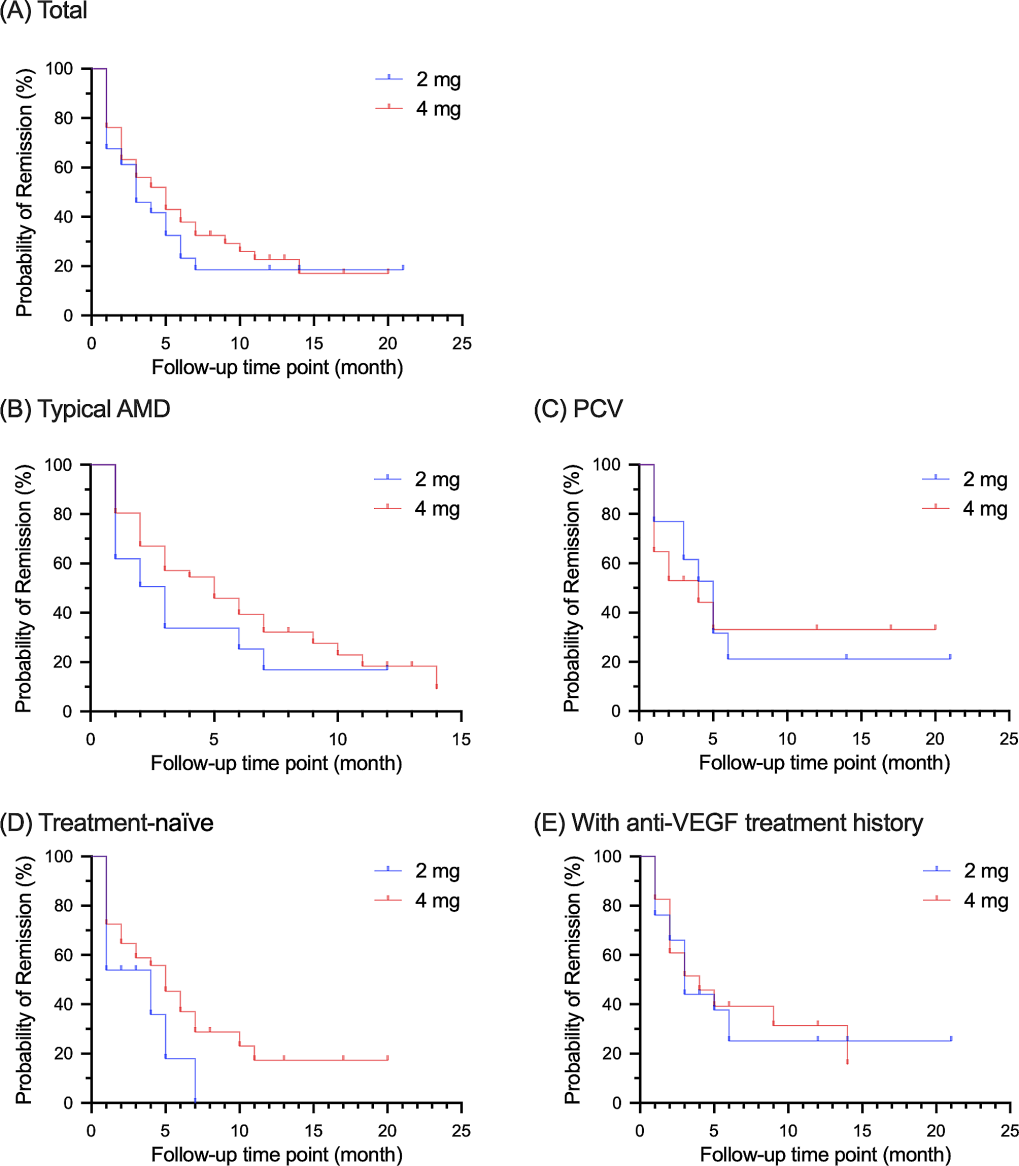
Survival curves of the remission intervals in the 2 mg group and the 4 mg group (A) Analysis of all the patients (B) and (C), analyses of subgroups divided by subtypes (D) and (E), analyses of subgroups divided by medical history AMD = age-related macular degeneration, PCV = polypoidal choroidal vasculopathy. PCV is a neovascular AMD subtype, and here typical AMD refers to type 1, 2 and 3 macular neovascularization in neovascular age-related macular degeneration

There were 97 eyes from 97 patients (if both eyes were available in one patient, the left eye was selected) included for survival analyses. The median remission interval was 5 months (95% CI = 2.962–7.038 months) in the 4 mg group, 2 months longer than the 3 months (95% CI = 1.154–4.846 months) in the 2 mg group (*P* = 0.452, Table 3). The relatively better performance of the aflibercept 4 mg group was also reported by the Cox regression (HR = 0.863, 95% = 0.492–1.513, *P* = 0.607). The survival curves showed that the benefit was mainly obvious between the 5th month and the 10th month (Fig. 2A).

**Table 3:** Comparison of remission intervals between patients receiving aflibercept 2 mg and aflibercept 4 mg

Remission intervals (months)		Dose	Kaplan-Meier test (Log-rank)		Cox regression^#^	
			Median time (months, 95% CI)	*P* value	HR (95% CI)	*P* value
Total		2 mg	3 (1.154–4.846)	0.452	0.000 (reference)	0.607
		4 mg	5 (2.962–7.038)		0.863 (0.492–1.513)	
Subtype	Typical AMD	2 mg	3 (1.286–4.714)	0.252	0.000 (reference)	0.618
		4 mg	5 (2.398–7.602)		0.833 (0.407–1.707)	
	PCV	2 mg	5 (3.120–6.880)	0.915	0.000 (reference)	0.527
		4 mg	4 (0.313–7.687)		0.707 (0.241–2.072)	
Treatment history	Naïve	2 mg	3 (1.987–4.013)	0.896	0.000 (reference)	0.967
		4 mg	4 (1.059–6.941)		0.982 (0.406–2.373)	
	Treated	2 mg	4 (-)	0.146	0.788 (0.370–1.678)	0.537
		4 mg	5 (3.206–6.794)			

HR = hazard ratio, AMD = age-related macular degeneration, PCV = polypoidal choroidal vasculopathy. PCV is a neovascular AMD subtype, and here typical AMD refers to type 1, 2 and 3 macular neovascularization in neovascular AMD

^#^Cox regression was controlled by age, gender, subtype, baseline central macular thickness, baseline pigmental epithelium detachment height, and medical history (treatment-naïve [naïve] or previously receiving anti-vascular endothelium growth factor treatment [treated])

When stratified by the subtype, we found that the benefits of elongated remission interval from aflibercept 4 mg were contributed by eyes with typical AMD rather than PCV. These benefits also gradually degraded as the number of injections increased. When stratified by the treatment history, a weak one-month-benefit of double-dose aflibercept were found in both treatment-naïve patients (HR = 0.982, 95% CI = 0.406–2.373, *P* = 0.967) and patients with anti-VEGF history (HR = 0.788, 95% CI = 0.370–1.678, *P* = 0.537).

### Number of injections

As Table 1 presented, among all enrolled patients, more injections were needed in the aflibercept 2 mg group than in the 4 mg group (3.644 ± 1.670 versus 4.286 ± 2.334, *P* = 0.151) within 12 months.

Generally, patients with PCV received more injections than patients with nAMD, and patients with anti-VEGF history needed more treatments than treatment-naïve patients (Table 4). Even stratified by subtype or medical history, patients with aflibercept 4 mg treatment still needed fewer injections in all subgroups (AMD or PCV, and treatment-naïve or previously treated).

**Table 4:** Comparisons of injections within the first 12 months between the 2 mg group and the 4 mg group

Injections		2 mg	4 mg	*P* value
Subtype	Typical AMD	3.773 ± 1.998	3.611 ± 1.698	0.722
	PCV	5.154 ± 2.672	3.737 ± 1.628	0.072
Medical history	Naïve	4.769 ± 2.522	3.660 ± 1.478	0.151
	Treated	4.000 ± 2.225	3.615 ± 2.002	0.532

There were 35 eyes from 34 patients in the 2 mg group and 73 eyes from 63 patients in the 4 mg group

HR = hazard ratio, AMD = age-related macular degeneration, PCV = polypoidal choroidal vasculopathy. PCV is a neovascular AMD subtype, and here typical AMD refers to type 1, 2 and 3 macular neovascularization in neovascular AMD. Naïve = treatment-naïve, treated = previously receiving anti-vascular endothelium growth factor treatment

^#^Paired-sample *t* test. Otherwise tested by independent-sample *t* test

**Fig. 3:**
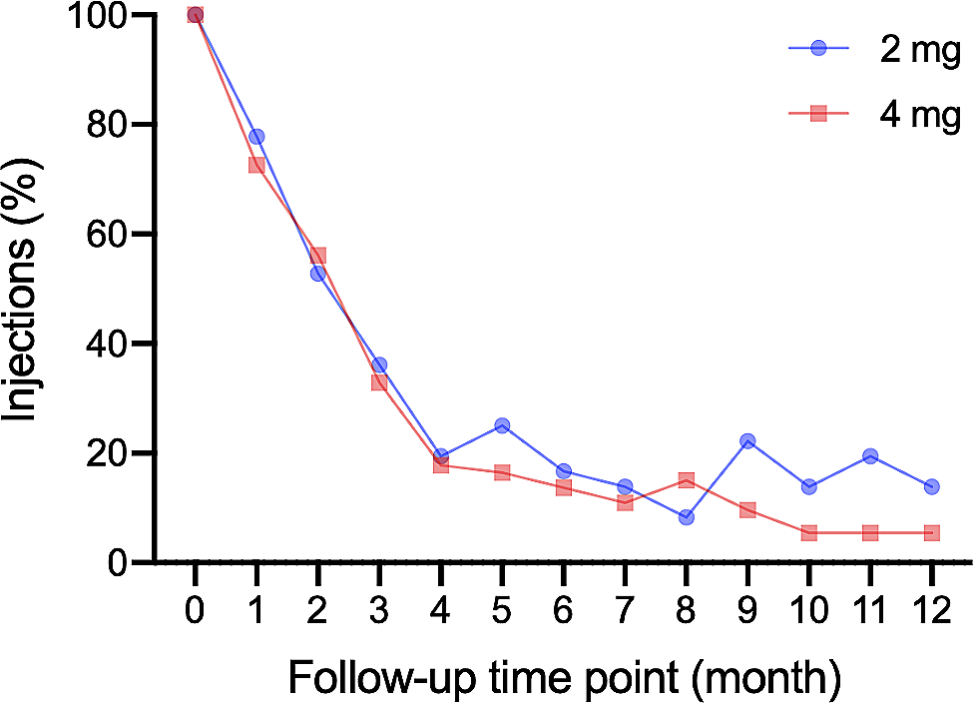
Injections in two dose groups during the 12-month follow-ups There were 35 eyes received the aflibercept 2 mg treatment and 73 eyes received the 4 mg treatment

When studying the percentages of eye receiving injections at each follow-up time point (Fig. 3), two groups had the percentages fall sharply and simultaneously until the 4th month. After that, the 4 mg group had it kept going down and stabilized around 5%, but the 2 mg group still had the injection percentages fluctuating between 10 and 20%. These results were consistent with longer remission intervals with aflibercept 4 mg treatment in the survival analyses.

### 2 Mg versus 4 mg self-control comparison

A total of 19 eyes received aflibercept 2 mg first and then aflibercept 4 mg treatment. However, 7 were excluded from self-control analyses due to the different follow-up duration of the two treatment regimens. The remaining 12 eyes were analyzed, but CMT/PED height values from another 5 eyes were missed due to serious exudation beyond measurement. These were 4 (33.33%) female patients and six left eyes (50.00%). Only two of them were diagnosed with PCV (16.67%).

No benefits of remission intervals were gained from the aflibercept 4 mg treatment, nor less exudation recurrence (Table 5). Compared with the aflibercept 2 mg group, tendencies of more CMT reduction (larger absolute value of CMT reduction) but less PED height reduction were observed in the 4 mg group, which were consistent with the general performance of the 4 mg treatment. In addition, comparable numbers of injections were given between the aflibercept 2 mg treatment and the 4 mg treatment (2.917 ± 1.832 versus 3.417 ± 2.151, *P* = 0.615).

**Table 5:** Self-comparisons of the clinical effects of the 2 mg group and the 4 mg group

Outcomes		2 mg	4 mg	*P* value
Cumulative remission interval (months)		4.4187 ± 4.582	3.417 ± 4.078	0.305^#^
Longest remission interval (months)		4.167 ± 4.609	3.083 ± 3.965	0.333^#^
CMT change (µm)^§^		-43.000 ± 101.913	-58.000 ± 89.792	0.551^#^
PED height change (µm) ^§^		-66.000 ± 139.944	10.857 ± 56.925	0.326^#^
Either IRF or SRF	No	3	2	1.000^*^
	Yes	9	10	
IRF only	No	6	7	1.000^*^
	Yes	6	5	
SRF only	No	8	7	1.000^*^
	Yes	4	5	
Number of injections (within 12 months)		2.917 ± 1.832	3.417 ± 2.151	0.615^#^

IRF = intraretinal fluid, SRF = subretinal fluid, Exudation = IRF and/or SRF

^#^Paired-sample *t* test

^*^Related-samples McNemar test (exact significances were displayed for these tests)

^§^Only 7 study eyes were included due to missing values

### Safety summary

No markedly ocular adverse event (AE), such as increased-IOP events, endophthalmitis in study eye or fellow eye, was not found in two groups. No systemic AE, such as abnormal systolic/diastolic blood pressure, abnormal heart rate, abnormal body temperature, cardiovascular stroke, or even fatal outcomes, was found in two groups, either.

## Discussion

This retrospective study aimed to investigate the clinical effects of aflibercept 4 mg treatment in a real-world setting. Results showed that aflibercept 4 mg treatment had advantages over the 2 mg group in anatomic gains, such as stable CMT reduction and more IRF resolution. However, a tendency of higher SRF incidence was also observed in the aflibercept 4 mg group. Compared to the aflibercept 2 mg treatment, this double-dose treatment can also defer the median remission interval and recurrence by 2 months, and reduced the injection needed during the one-year follow-ups with the PRN regimen. No additional visual gains were obtained with the 4 mg treatment.

Similar to other high-dose anti-VGEF studies [3, 13], anatomic gains were seen in the aflibercept 4 mg group with a PRN regimen in 12 months. The lower IRF risk, but not SRF, was observed in the 4 mg group (adjusted OR = 0.433, 95% CI = 0.183 to 1.022, *P* = 0.056, Table 2), which could be explained by the greatest and most rapid nature of IRF resolution with anti-VEGF treatment [14]. Also, it was refractory IRF rather than refractory SRF that associated with a higher risk of fibrosis and atrophy [15]. Although a higher SRF incidence (OR = 1.539, 95% CI = 0.755 to 3.139, *P* = 0.236) was also observed with the aflibercept 4 mg regimen, the discrepancy did not eliminate the advantage of aflibercept 4 mg regimen in anatomic gains. The CMT reduction but no PED height reduction was detected in the 4 mg group as well, attributed to PED’s least response to the anti-VEGF treatment [14]. Due to the effect plateaus [13], ceiling effect [1, 13]and less baseline CMT/PED height in the 4 mg group (Table 1), CMT/PED height reduction of both the 2 mg and the 4 mg treatment tend to diminish as time went by and less PED height reduction in the 4 mg group. However, an advantage of CMT reduction in the 4 mg group was observed (regression coefficient = -25.242 μm, 95% CI = -90.024 μm to 39.539 μm, *P* = 0.445, Table 2), indicating the potency of CMT reduction with 4 mg treatment. Thus, the aflibercept 4 mg was promising in the anatomic restoration.

However, different from visual acuity sustaining with high-dose therapy in treatment-resistant nAMD [13], there was a significantly higher likelihood of visual preservation with the aflibercept 2 mg regimen in this study. This might be explained by the relatively worse baseline visual acuity [16] in the 4 mg group. There were over 50% of study eyes with baseline visual acuity worse than 20/63 (LogMAR > 0.5) as well. A significant visual improvement could not be warranted [17]. Furthermore, as patients in the 4 mg group had a long course of nAMD, as about 2/3 of study eyes had received an average of over 10 anti-VEGF injections, less remaining healthy photoreceptors and therefore vision loss could not be avoided [18, 19]. As we also observed a more stable reduction in retinal thickness [20] and retinal fluid volumes [21] in the 4 mg group, especially better IRF solution [22], which all contributed to better visual prognosis, greater visual performance was expected with active aflibercept treatments.

Compared to the 2 mg group, fewer injections and extended remission intervals were found in the 4 mg group. The median remission interval in the 4 mg group was 5 months, 2 months longer than that in the 2 mg group (Table 4; Fig. 2). This trend was more obvious in study eyes diagnosed as typical AMD but weak in eyes with PCV, probably resulting from the complex and resistant nature of PCV lesions [23]. However, when considering the first 12-month follow-ups, about 1.5 more injection reduction in the 4 mg group was found in study eyes with PCV, indicating the advantage in injection burden with the 4 mg treatment in both subtypes. The injection reductions were also found in patients with and without anti-VEGF history. These were different from a previous study reporting high injection frequency required in high-dose anti-VEGF treatment in nAMD, including the 2 mg ranibizumab [3] and 3 mg aflibercept [13]. The strength of aflibercept, especially the aflibercept 4 mg treatment, in reducing treatment burdens should be highlighted in real-world settings. As elongated remission intervals were also observed in treatment-naïve eyes, the double-dose aflibercept treatment could be chosen in these cases to relieve the nAMD disease burden to some extent.

Though the advantages of the 4 mg treatment in both anatomic gains and treatment burden relieves were detected in the general patients, they were not supported by the self-control analyses. This may be explained by (1) the small sample size in the self-control analyses, (2) the late disease course during the 4 mg period, as all patients got treatment switched from 2 mg to 4 mg, (3) the treatment-resistant nature of these eyes [1], and (4) consistent application of aflibercept and drug switch might be needed [24]. Thus, switching to 4 mg treatment may not recommend for patients who had long disease durations and responded poorly to the aflibercept 2 mg treatment.

For safety considerations, no markedly increased-IOP events, endophthalmitis, or cardiovascular adverse events were found in two groups. Considering the theoretical IOP-increasing-risk of higher volume injection, the aflibercept 4 mg treatment was still not recommended for patients with glaucoma or high IOP.

The limitations of this study included its retrospective nature, short follow-up duration, the relatively small sample size, and the only recruitment of Chinese population. A larger sample size, longer-term follow-up periods, and higher racial diversity are warranted in the future, as it would provide more comprehensive insights into the sustained efficacy and safety of double-dose aflibercept treatment. A prospective randomized controlled trials should also be expected. Alternatively, our real-world setting increased the clinical strength of this study. In addition, we recruited not only treatment-naïve patients, but also patients with anti-VEGF treatment history, even those experiencing both aflibercept 2 mg treatment and 4 mg treatment. We believe that our study adds to the information being considered by retinal specialists for further application of aflibercept in nAMD. Additional anatomic gains could be obtained by some patients starting with or switching to the aflibercept double-dose regimen. And double-dose usage may reduce the injection needed to a certain extent. Since the aflibercept 4 mg treatment used in this study only had the antibody volume increased (to 100 µL) but not the concentration increased, more promising clinical effects with further high-dose and high-concentration aflibercept (8 mg, 70 µL) can be expected. As these high-dose products still have a long way to go to be commercially available and covered by health insurance in different countries and areas, the current product can be applicated with double dosage in some patients at this moment.

## Conclusions

In conclusion, compared to the aflibercept 2 mg treatment in nAMD, tendencies of anatomic gains and relieving treatment burden were brought by the aflibercept 4 mg treatment. This study may have additional importance, given the further application of high-dose aflibercept in real-world settings.

### Electronic supplementary material

Below is the link to the electronic supplementary material.

Supplementary Material 1

## Data Availability

All data generated or analyzed during this study are included in this published article and its supplementary information files.
