# Supplementary material for: Double-dose investigation of aflibercept in neovascular age-related macular degeneration (DIANA): a real-world study
Source: BMC Ophthalmol. 2024 May 17;24:215. doi: 10.1186/s12886-024-03476-9 (PMC11100152; doi:10.1186/s12886-024-03476-9)
Supplement: Supplementary file 1 — Supplementary Material 1 [file 12886_2024_3476_MOESM1_ESM.docx]

**Supplementary Materials**

| **Table S1**. Illustration of the definition of “at-least-3-month-interval before the study baseline”, taking one patient’s treatment schedule as an example. | | | |
| --- | --- | --- | --- |
| Date | Anti-VEGF treatment | Dose | If included in this study |
| December, 24, 2018 | Conbercept | Standard | No |
| January, 28, 2019 | Conbercept | Standard | No |
| February, 25, 2019 | Ranibizumab | Standard | No |
| March, 25, 2019 | Ranibizumab | Standard | No |
| April, 21, 2019 | Aflibercept | Standard | No (only 1-month interval from the last ranibizumab injection) |
| May, 20, 2019 | Aflibercept | Standard | No (only 1-month interval from the previous un-included injection) |
| June, 17, 2019 | Aflibercept | Standard | No (still only 1-month interval from the previous un-included injection) |
| October, 14, 2019 | Aflibercept | Standard | Yes (after nearly 4-month washout period from the previous un-included injection) |
| December, 16, 2019 | Aflibercept | Standard | Yes |
| January, 13, 2020 | Aflibercept | Standard | Yes |
| May, 11, 2020 | Aflibercept | Standard | Yes |
| August, 31, 2020 | Aflibercept | Double | Yes (after over 3-month washout period from the last 2 mg dose treatment) |
| September, 28, 2020 | Aflibercept | Double | Yes |
| January, 4, 2021 | Aflibercept | Double | Yes |


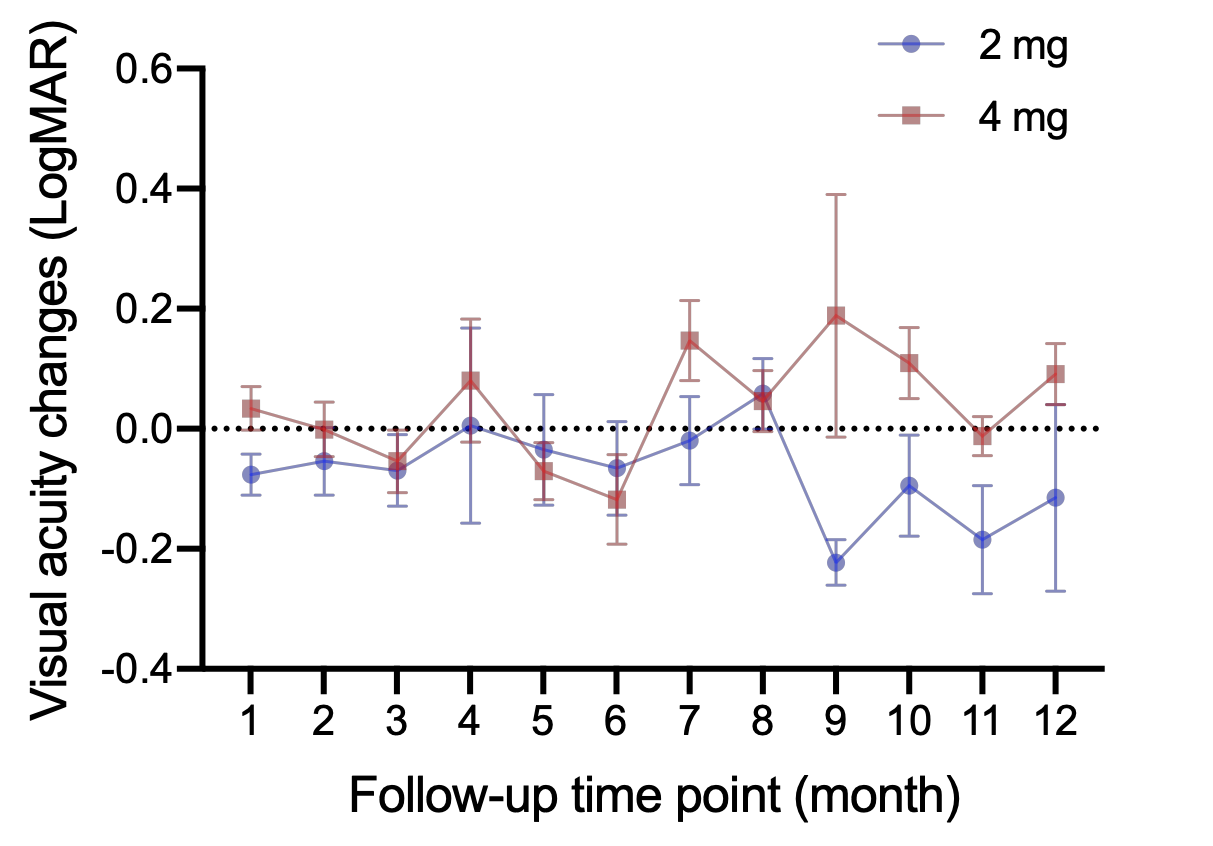


**Figure S1.** Visual acuity changes in two dose groups during the first 12 months.

Data were presented as mean ± standard error.
